# Methodological Problems With Online Concussion Testing

**DOI:** 10.3389/fnhum.2020.509091

**Published:** 2020-10-01

**Authors:** Jameson Holden, Eric Francisco, Anna Tommerdahl, Rachel Lensch, Bryan Kirsch, Laila Zai, Alan J. Pearce, Oleg V. Favorov, Robert G. Dennis, Mark Tommerdahl

**Affiliations:** ^1^Cortical Metrics LLC, Carrboro, NC, United States; ^2^Lucent Research, Denver, CO, United States; ^3^College of Health Science and Engineering, LaTrobe University, Melbourne, VIC, Australia; ^4^Department of Biomedical Engineering, The University of North Carolina at Chapel Hill, Chapel Hill, NC, United States

**Keywords:** reaction time, reaction time variability, online cognitive testing, online concussion testing, intraindividual reaction time variability, concussion, concussion testing

## Abstract

Reaction time testing is widely used in online computerized concussion assessments, and most concussion studies utilizing the metric have demonstrated varying degrees of difference between concussed and non-concussed individuals. The problem with most of these online concussion assessments is that they predominantly rely on consumer grade technology. Typical administration of these reaction time tests involves presenting a visual stimulus on a computer monitor and prompting the test subject to respond as quickly as possible via keypad or computer mouse. However, inherent delays and variabilities are introduced to the reaction time measure by both computer and associated operating systems that the concussion assessment tool is installed on. The authors hypothesized systems that are typically used to collect concussion reaction time data would demonstrate significant errors in reaction time measurements. To remove human bias, a series of experiments was conducted robotically to assess timing errors introduced by reaction time tests under four different conditions. In the first condition, a visual reaction time test was conducted by flashing a visual stimulus on a computer monitor. Detection was via photodiode and mechanical response was delivered via computer mouse. The second condition employed a mobile device for the visual stimulus, and the mechanical response was delivered to the mobile device's touchscreen. The third condition simulated a tactile reaction time test, and mechanical response was delivered via computer mouse. The fourth condition also simulated a tactile reaction time test, but response was delivered to a dedicated device designed to store the interval between stimulus delivery and response, thus bypassing any problems hypothesized to be introduced by computer and/or computer software. There were significant differences in the range of responses recorded from the four different conditions with the reaction time collected from visual stimulus on a mobile device being the worst and the device with dedicated hardware designed for the task being the best. The results suggest that some of the commonly used visual tasks on consumer grade computers could be (and have been) introducing significant errors for reaction time testing and that dedicated hardware designed for the reaction time task is needed to minimize testing errors.

## Background

There are numerous online tests that are routinely used for assessments of individuals with concussion and/or used in concussion research, and the majority of these assessment tools have components that specifically address the reaction time and/or reaction time variability of the individual that is being tested. However, these contemporary concussion computerized online assessment tools predominantly rely on whatever computer systems that they are downloaded and run on. Administration of the reaction time test by these online assessments typically use the computer's monitor and mouse to deliver a stimulus (such as a character on the monitor) and receive a response (button press of the computer mouse), respectively. As the prevalence of online concussion testing that relies on consumer grade computers has increased while the utilization of laboratory research tools has decreased, the authors sought to determine if that could have an impact on performance and accuracy of the reaction time test.

A study by Woodley et al. (2013) postulated that as a human race, we are getting “dumber.” The basic premise of the study was that reaction times are getting slower, and that this contradicts a number of other studies that had demonstrated that, based on performance on IQ-tests, we are actually getting a bit smarter. The purpose of this report is not to weigh in on whether or not humans are getting dumber as a species, but rather to focus on the accuracy of reaction time testing, how it has changed historically and if these changes are the result of altered scientific methodology that could be inadvertently leading to inaccurate scientific findings in the literature.

**Figure 1:**
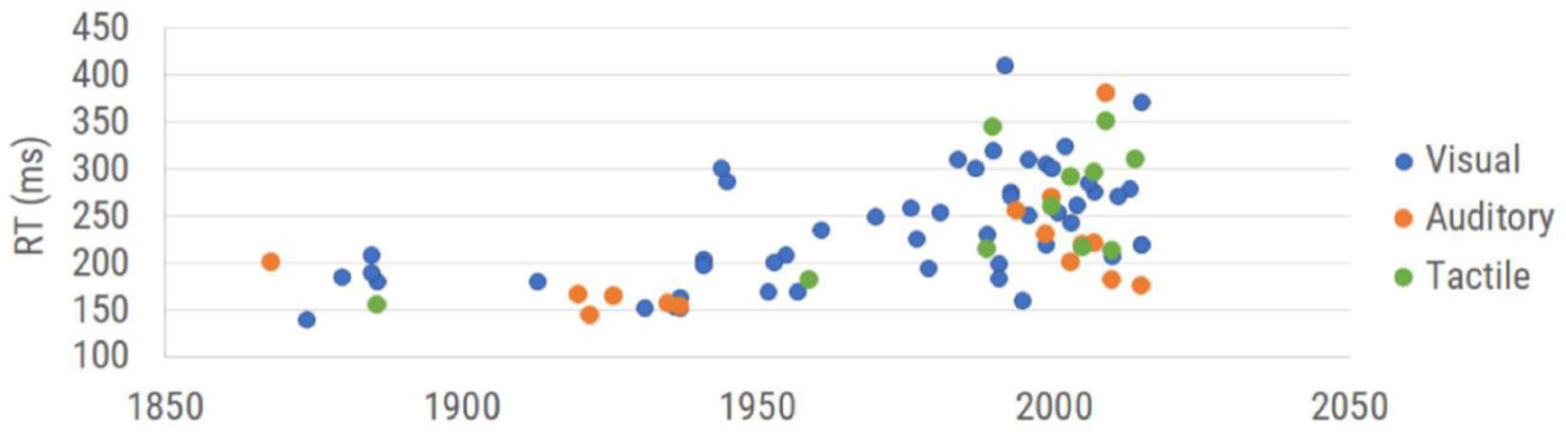
Results of reaction time testing reported in the literature. Reaction time values are categorized by modality of measurement (visual reaction time, auditory reaction time, and tactile reaction time) and plotted in the corresponding year collected. See Table 1 (see Appendix A: Supplementary Tables) for complete references used to obtain each data point.

There have been many reaction time studies over the past 150 years. The graph in Figure 1 is a summary of the data points obtained from healthy subjects across a collection of those publications. Each plotted data point is the overall average obtained for healthy controls in each of the selected studies (which is inclusive of the 16 studies used by Woodley et al., 2013 plus additional reports that focused on reaction time). The data demonstrate not only an upward drift of reaction time, but a larger range of reaction times, with the progressive degradation of reaction time appearing to begin in the 1970s and 1980s. Thus, the question that the authors think should be asked is not whether we are getting worse at reaction time testing, but could there be inconsistencies introduced by the reaction time testing itself?

Note that while early reaction time studies (between 1850 and 1950) demonstrated human performance in the 150–200 msec range, the range reported post-2000 extends from 150 to 400 msec. This enormous shift suggests either a very different population that is being tested or a very different strategy for measuring reaction time. Since the dumbing down of the human species is really not something that the authors believe can be accurately measured (other than observations of how much time some generations spend utilizing social media), we targeted addressing the actual methods that were being used. Casual observation of the data plotted in Figure 1 suggests that the methods underlying the represented scientific literature are becoming much more variable and consequently, less accurate.

Reports in the literature describing and/or utilizing reaction time tests date back to the nineteenth century. In 1849, Hermann von Helmholtz used electric stimulation to investigate nerve conduction velocity, first examining the conduction velocity in the legs of frogs before modifying his methods to accommodate human subjects. Helmholtz stimulated the skin at two separate locations and measured the time required for the human subject to respond via hand signal to the stimulation at each location. By measuring the distance between the two points of stimulation and the difference in their associated reaction time, he deduced a fairly accurate estimate of nerve conduction velocity. Donders expanded on the concept to include central as well as peripheral nervous system processing in which he stimulated either skin, eye or ear and had the subjects respond with their hands (Donders, 1868, 1969). Time recorded was based on a chronograph, and although the early methods may seem crude or cumbersome by contemporary methods, the results from these early experiments inspired numerous investigations utilizing reaction time over the subsequent 150 plus years. The methods used by Merkel, visual stimulation and tactile response (Merkel, 1885), are still commonly used due to the simplicity of implementation, though it is noteworthy that the individual data obtained by Merkel ranged between 152 and 201 msec, which is not the case for most contemporary studies.

Many of the studies spanning the 150-year time frame investigated changes in reaction time that resulted from a number of neurological disorders or insults. For example, reaction times have been demonstrated to be altered by changes introduced by neurological insults such as TBI/mTBI (Ruesch, 1944; van Zomeren and Deelman, 1976; MacFlynn et al., 1984; Stuss et al., 1989; Ponsford and Kinsella, 1992; Collins and Long, 1996; Zahn and Mirsky, 1999; Warden et al., 2001; Collins et al., 2003; Sarno et al., 2003; Willison and Tombaugh, 2006; Niogi et al., 2008; Gould et al., 2013; Eckner et al., 2015), PTSD (Ruesch, 1944), pharmaceuticals (Edwards and Cohen, 1961; Ancelin et al., 2006), aging (Benton, 1977; Sherwood and Selder, 1979; Fozard et al., 1994; Lajoie and Gallagher, 2004; Der and Deary, 2006; Zhang et al., 2011; Woods et al., 2015), dementia (Ancelin et al., 2006), Parkinson's (Evarts et al., 1981; Goodrich et al., 1989), schizophrenia (Schwartz et al., 1989), ADHD (Meere et al., 1992; reviewed in Tamm et al., 2012; Puts et al., 2017), sleep deprivation (Lorenzo et al., 1995), caffeine (Cheney, 1934), alcohol (Hernández et al., 2006), autism spectrum disorders (Puts et al., 2014; Ferraro, 2016), and diabetes (Patil and Phatale, 2015). The widespread utilization of the reaction time test across many decades of research and its utility in many different clinical and clinical research venues led us to ponder how the accuracy of this measure might have changed historically. Contemporary users of the reaction time metric might assume that using modern and faster computer technology automatically leads to more accurate reaction time measures. The fallacy of this assumption is that the modern computer technology commonly deployed for reaction time testing is not designed to be laboratory equipment, which causes the accurate timing of external events to suffer. Laboratory equipment of the mid-nineteenth century was specifically designed for laboratory use and was probably as accurate at performing reaction time testing as many of today's computer-based reaction time tests, if not more so.

The question we sought to address was whether or not modern computing methods introduce problems to the reaction time measure. There are inherent delays predicted to be introduced by both software and hardware. Many contemporary reaction time tests are administered through a computer program that calculates the time elapsed between stimulus delivery and the subject response (typically the click of a mouse). The majority of these tests use either a visual (e.g., screen flash) or auditory (loud beep) stimulus, as these stimuli can be delivered using commodity-grade human-computer interfaces such as computer monitors, mice, keyboards, and touch screens. Reaction time tests that employ a different mode of stimulation (such as a tactile stimulus) require additional hardware, which may be connected to the test computer by a physical or wireless connection. The reaction time test is contingent on the CPU timing accuracy of the testing computer, which can vary based on which programs are running in the background and the inherent processing speed of the chip. Also of great concern is the operating system (OS) timing cycle and task priority structure, which typically manages many tasks, including system overhead unrelated to the reaction time test in progress. While this division of attention is seldom apparent to the user, it typically introduces delays of ~15 msec, or more when the computational demand is high, such as in the presence of malware or background tasks, or as a result of widely employed networking prioritization such as “audio prioritization.” In this case, the delays in other functions can even become clearly apparent to the user, being on the order of hundreds of milliseconds or more. Even at its minimum, this CPU latency is typically between 2 and 20 msec, which will significantly and ambiguously alter reaction time test results. In addition to OS latency, different device driver firmware can introduce latencies differing by tens of milliseconds or more between drivers, even with identical hardware (Plant and Turner, 2009). These computer hardware and software variations can introduce variable timing delays of up to 100 msec (Neath et al., 2011).

Reaction time tests suffer from latencies that are introduced at points aside from core processor timing in their protocols as well. For example, these can be introduced by the commodity human interface peripherals. USB and wireless mice and keyboards introduce latency in their communication protocols at several points, including pre-transmission buffering, transmission, and post-transmission buffering before transfer to the CPU for processing. Most computer screens have a refresh rate of 60 Hz, and a screen flash can occur up to 17 msec before or after the “stimulus delivery” time that is recorded by the CPU. Touch screens on both mobile and desktop devices have a built-in latency related to the sensing mechanism, usually by capacitance. For smooth operation, touch sensing requires a certain amount of signal processing both in hardware and software or firmware, because touch signals typically involve a certain amount of “integration” at or near the sensor. This is necessary both for noise mitigation, to eliminate spurious signals, as well as to detect the proximity of a finger or stylus by capacitive coupling. For example, capacitive sensing can be done in several different ways, but these all invariably involve the rate of charge or discharge of a capacitor which is modified by the proximity of a finger or stylus that changes the value of the capacitor being charged, and thus the RC time constant, by a few percent. Styli may be standardized for a specific device, but fingers are not, and thus it is necessary to set thresholds and make decisions in firmware whether or not a touch event has occurred, or not, for a wide range of contact conditions. All of this integration and processing takes time, even when done by distributed processing. The same arguments can be modified and applied to older force sensing screen interfaces which had the added computational burden of calculating a force centroid to determine where force was being exerted on the plane of the display, and similarly for any other touch sensing strategy such as resistive or others. Layered on top of this is the firmware task of interpreting what type of touch is being detected, whether there are one or multiple touch points. Once the signal has been cleaned, filtered, and interpreted by the peripheral touch sensing device, it can then be placed in the communication buffer, where it waits its turn for CPU priority. Because of all the variables involved, and different strategies employed by touch sensing peripheral hardware, it is not possible to calculate the hardware latency. As a result, current “lag” from touch screen-to-display varies from 50 to 200 msec (Ng et al., 2012).

The objective of this study was to determine if there are significant and measurable differences introduced to reaction time measures that are collected with different types or categories of hardware currently used in commercially available reaction time tests. More specifically, how would a visual reaction time test performed on a computer laptop or mobile device with consumer grade hardware (most common method) compare with a tactile reaction time test delivered with laboratory grade hardware (less commonly performed). If equipment and/or operating systems do in fact introduce errors into the reaction time test, then it would be hypothesized that different methods requiring significantly different hardware or software would generate very different reaction times and reaction time variability for individuals taking the reaction time test. In order to directly investigate the differences introduced by various testing strategies, the human element was removed from this study and automated/dedicated hardware was used to perform the reaction time tasks. Four modes of reaction time testing were evaluated robotically to compare the potential contributions of different user interfaces to the reaction time test.

## Materials and Methods

Experiments were conducted with four different conditions in order to observe results obtained with variable stimulus and response protocols for a reaction time test with a non-human interface. The stimuli delivered were visual/optical (simulation of a visual stimulus) with a mobile device, visual/optical with a computer monitor, and tactile/mechanical (simulation of a mechanical stimulus) with a dedicated hardware device (mechanical stimulus delivered with the Brain Gauge; Cortical Metrics, LLC). The Brain Gauge is a commercially available tactile stimulator that both delivers vibrotactile stimuli and can be used as a response device and was well-suited for this study. Originally developed for research laboratory use with good resolution, the Brain Gauge maintained technical specifications as it evolved to a commercial product. The response methods used were tactile/mechanical via touchscreen (simulation of finger press on touch screen), tactile/mechanical via computer mouse (simulation of finger response via computer mouse), and tactile/mechanical via dedicated hardware device (simulation of finger response via Brain Gauge).

### Apparatus and Device Setup

Four simulations were performed, and different configurations were used to deliver those simulations. In each case, simulation of an individual taking a reaction time test was performed by detection of a visual or mechanical stimulus via electronic switch to simulate stimulus detection and delivery of a mechanical stimulus to simulate a finger depressing a response device. The configurations were assembled with a standard breadboard. Each of the following tasks ran for *N* = 100 trials and each simulation was conducted with the same CPU (MacBook Pro 2017). Trials were performed for either a visual or mechanical stimulus simulation. Detection simulation was performed by mechanical response to either a computer mouse, a touchscreen or a dedicated device (Brain Gauge; Cortical Metrics, LLC).

### Simulation #1: Visual Stimulus and Response via Mobile Device

**Figure 2:**
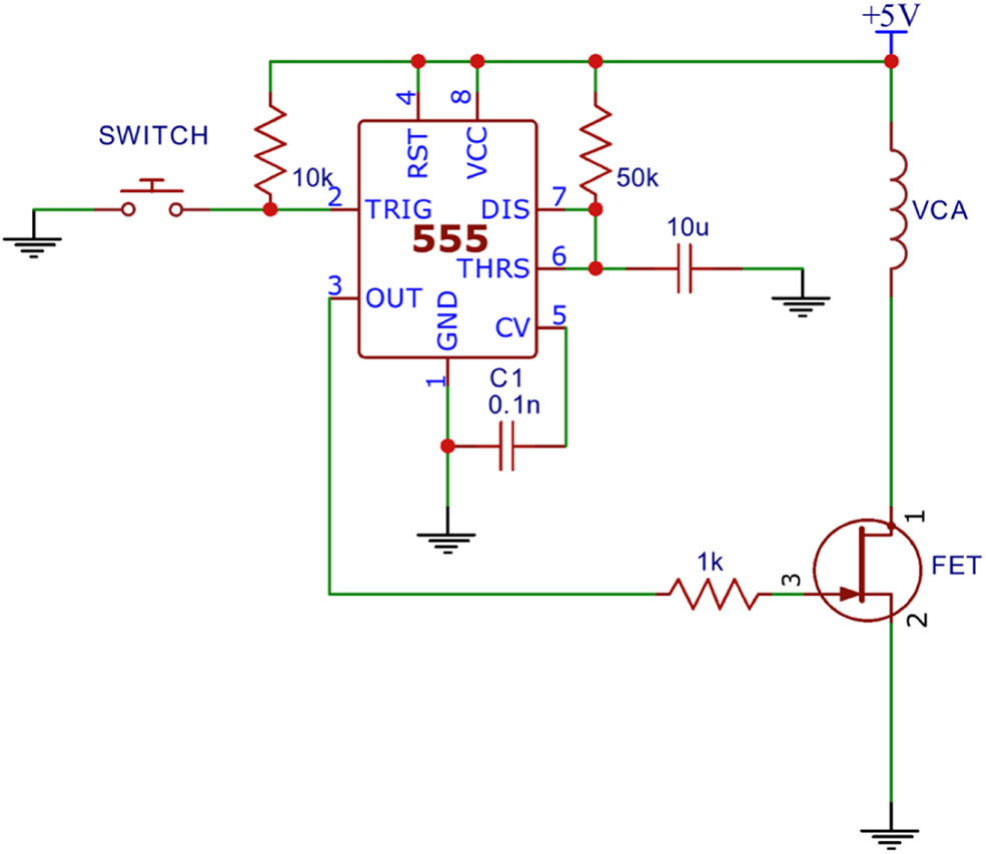
Dedicated mechanical apparatus.

An analog light sensor (Adafruit ALS PT19) was mounted 2 mm above a mobile touchscreen (Nextbit Robin, Android 7.1.1 “Nougat”), which was programmed to flash from black to white at random intervals (4–6 s). The light sensor was configured with a triggering threshold of 800 mV. In order to simulate the response interval of a reaction time test, a dedicated mechanical apparatus was designed that was triggered to respond automatically at a fixed interval (100 msec) after the flash was detected by the light sensor. The mechanical apparatus (Figure 2) was assembled on a standard breadboard and was comprised of a linear voice coil actuator (VCA), a 555 Timer configured for monostable output (110 ± 1 msec), an N-channel field-effect transistor (FET), and a 5 V power supply.

**Figure 3:**
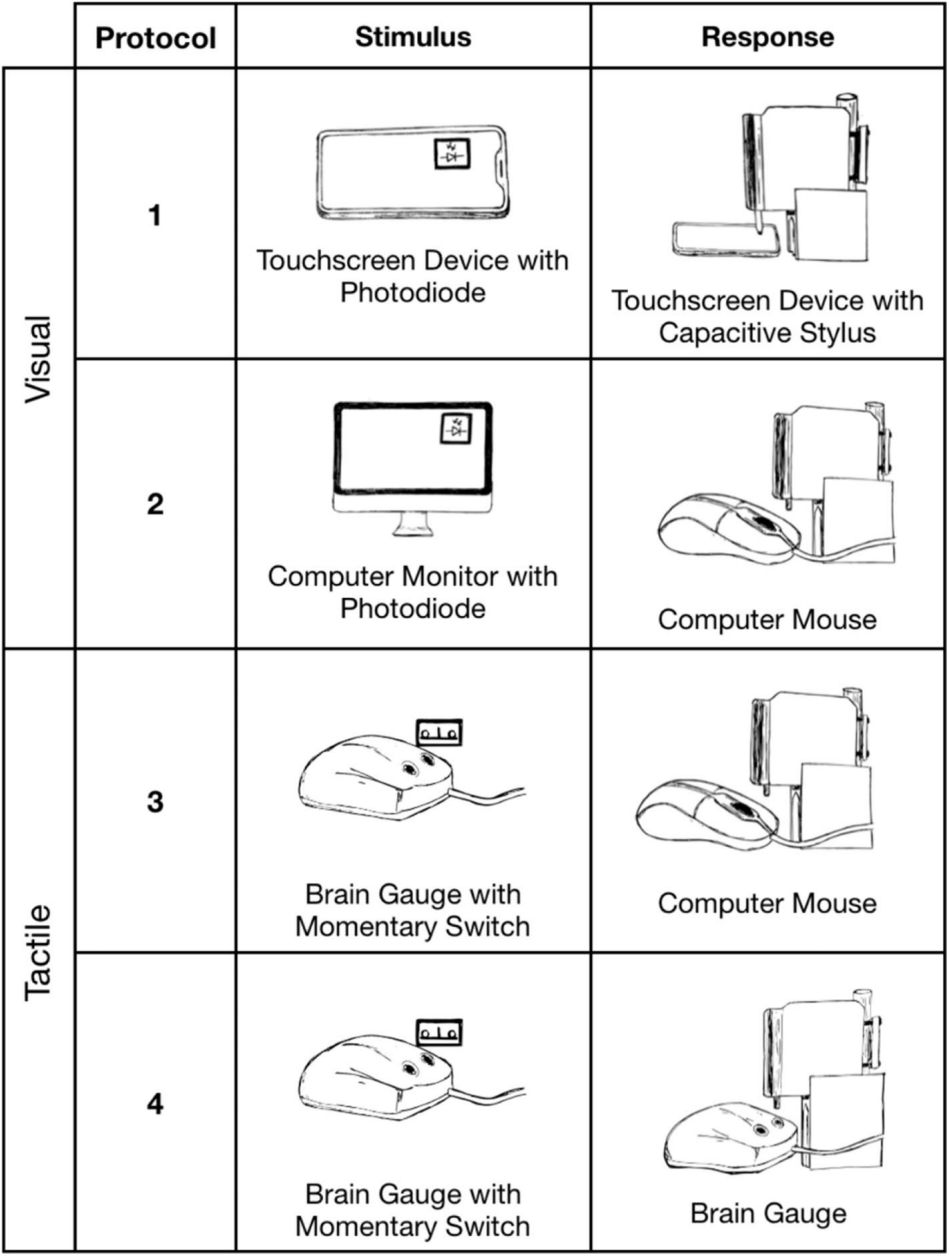
Reaction time simulations. Protocols 1 (Nextbit Robin) and 2 (MacBook Pro 2017) delivered mechanical stimuli in response to a visual stimulus. Protocols 3 and 4 (MacBook Pro 2017) delivered mechanical stimuli in response to a tactile stimulus.

A capacitive sensor was mounted to the VCA probe tip of the apparatus and placed 2.6 mm above the touchscreen in order to simulate a button press by a human (Protocol 1 of Figure 3). After the programmed delay the VCA would receive a 10 ms pulse from the 555 timer which caused the VCA to actuate. The capacitive sensor reached its expected triggering point (initial screen contact) at the middle point of the pulse, theoretically adding 5 msec to the 100 msec programmed delay. Thus, the expected true reaction time for the system was 105 msec.

### Simulation #2: Visual Stimulus and Response via Computer Mouse

The analog light sensor was placed 2 mm in front of an LCD monitor (60 Hz, 1080p, Dell) and was programmed to flash from black to white at random intervals (4–6 s). The light sensor was configured with a triggering threshold of 800 mV. The VCA of the mechanical apparatus was positioned directly above the left button of the USB computer mouse to simulate a subject's response. The mechanical response was simulated as in Simulation #1 and was used to simulate a finger press 100 msec after the flash triggered the light sensor. Expected true reaction time for the system was 105 msec.

### Simulation #3: Tactile Stimulus and Response via Computer Mouse

A mechanical switch (Cherry MX Red) was mounted above the probe tip of a tactile stimulator (Brain Gauge; Cortical Metrics, LLC) in order to detect a mechanical stimulus of a simulated reaction time test. A 1.5 mm stimulus was used to depress the switch above the actuation point and trigger the mechanical response simulator circuitry. The VCA probe tip of the mechanical apparatus was positioned above a computer mouse to simulate a subject's responding digit in a resting state. The stimulus pattern, programmed delay and switch response were identical to the conditions in Simulation #1. The mechanical apparatus from Simulation #1 was modified to simulate a controlled human reaction time of 100 msec after the mechanical switch was triggered. Expected true reaction time for the system was 105 msec.

### Simulation #4: Tactile Stimulus and Response With Dedicated Hardware Device

The mechanical detection system was arranged as in Simulation #3. The VCA probe tip was mounted above the response tip of the dedicated hardware reaction time device, depressing the device's tip by 1.5 mm. The VCA response was identical to the task of Simulation #3. The mechanical apparatus from Simulation #3 was used to simulate a controlled human reaction time of 100 ms after the mechanical switch was triggered. Expected true reaction time for the system was 105 ms.

## Results

Four simulated reaction time tests were performed. In each case a stimulus (visual or tactile) was delivered and detected electronically, and a response was made mechanically either via touchscreen, USB mouse or with a dedicated testing device (Brain Gauge; Cortical Metrics, LLC). The expected true reaction time for all four experimental groups was 105 msec. Results reported below have subtracted out the commanded 555 timer delay of 100 msec which was held constant using identical hardware for all testing protocols. A reported latency value of 5.0 msec would indicate the system achieved the expected results after adding back the 100 msec 555 timer delay.

**Figure 4:**
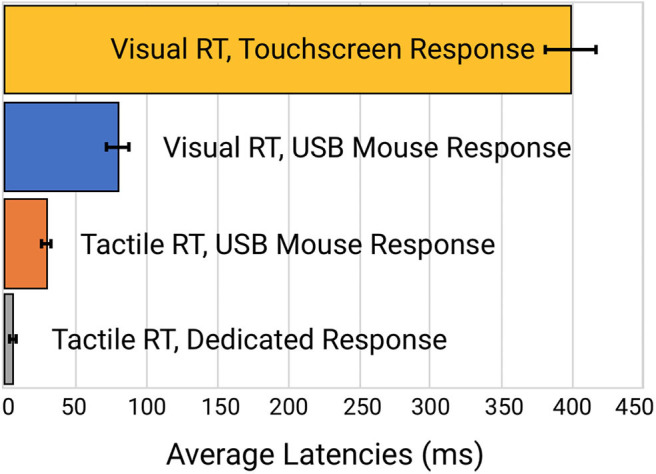
Comparison of the latency errors of four different methods of reaction time testing.

Reaction time to a simulated visual stimulus in which a touchscreen was used as the response device generated the highest latency of 399 ± 16.3 msec. When the same visual stimulus simulation was coupled with a response from a USB Mouse, reaction time latency was significantly improved to 80.1 ± 8.0 msec. Reaction time to a tactile stimulus simulation utilizing the same USB mouse for a response device demonstrated a latency of 30.7 ± 2.6 msec. Reaction time to the tactile stimulus simulation with response on the dedicated tactile device had the smallest latency error of 5.6 ± 0.25 msec. Average latency errors are plotted in Figure 4.

**Figure 5:**
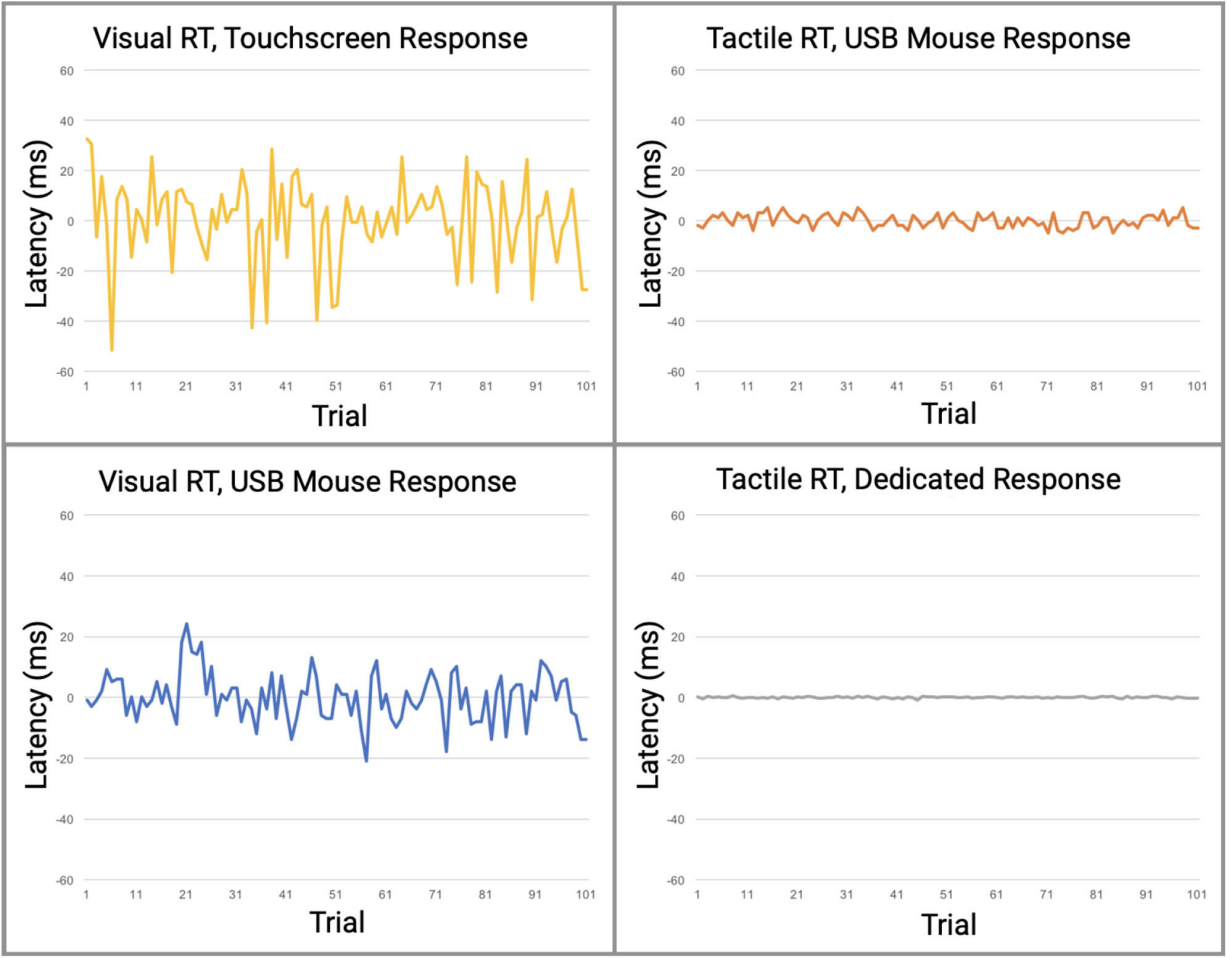
Direct comparison of data from the four reaction time testing methods with averaged offset subtracted. Raw data is plotted with offset of median latency subtracted, a technique used by many reaction time assessments to adjust for systematic latencies. All data is plotted on the same scale. The visual task simulations had significantly greater variabilities than the tactile simulations.

**Figure 6:**
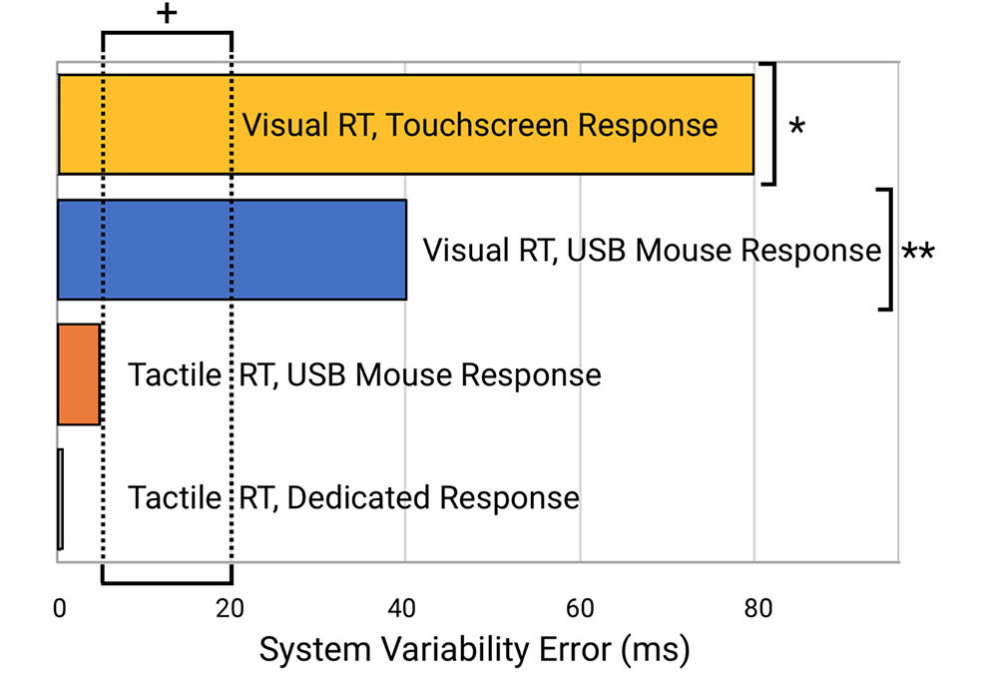
Comparison of system errors potentially introduced from the four different testing modalities. Note the indicated normative range of reaction time variability for human subjects (5–20 ms; indicated by “+”) is well below the errors introduced by the visual reaction time testing modalities (e.g., “*” for systems that use a visual stimulus and touchscreen device such as an iPad and “**” for systems that provide a visual stimulus and mouse or keyboard response with a desktop computer or laptop).

Perhaps more significantly than the latency on each task was the variability. In Table 2 (see Appendix A: Supplementary Tables), note the variability and range of latencies. This variability is prominently noticeable in Figure 5 which displays the data point-by-point and Figure 6 which directly compares system variability.

## Discussion

In this study, we demonstrated that reaction time testing, simulated robotically, shows profound differences in performance when stimuli are delivered either by visual or tactile modalities, and that there are significant differences in performance when the responses to those stimuli are delivered mechanically via either touchscreen, USB mouse, or a dedicated device that was designed for the task. In other words, a comparison was made in reaction time performance between consumer-grade computer interfaces and laboratory equivalent research tools with no human factors involved. The consumer-grade based testing that used visual stimuli demonstrated significant inaccuracies when compared to the tactile based testing. As demonstrated in Figure 5, relative contributions of latencies can be inferred from the various combinations of individual components. Protocols 1 and 2 have the highest overall latencies and variabilities and are the only protocols using a visual display. Since both the Nextbit Robin and Macbook Pro displays are clocked at 60 Hz, both have multiple processing cores, and given that all direct user input on both macOS and Linux is handled by kernel interrupts at the lowest level, differences in numerical computational power between the devices should not be a factor for such simple user input tasks. Therefore, the authors conclude that the primary contributor to the observed difference in latency between Protocols 1 and 2 is the touchscreen digitizer. Other touchscreen digitizers may exhibit less latency than observed in Protocol 1, but it is highly unlikely that any capacitive digitizer could outperform a simple USB mouse in a consumer-grade device. At best, the observed latencies for other 60 Hz touchscreen devices would approach those observed in Protocol 2.

The fundamental deficiency of commodity-grade computer interfaces for use in high-fidelity human performance research has been a concern for decades among researchers who value accuracy over simplicity. While there are a number of reports that demonstrate that many researchers have developed specialized laboratory equipment for the accurate measurement of human performance, a large and growing cohort of researchers are more frequently using less specialized equipment. From a high-altitude perspective, it appears that there was a time in the scientific study of human performance when researchers actually built and understood their instruments, tested and calibrated them carefully, knew their strengths and limitations, and factored these into the analysis of their results. But with the advent and ubiquity of low-cost commodity-grade human interface devices and displays designed to give the *perception* of smooth operation while being increasingly simple to use and low in cost, the use of these devices as if they were scientific-grade instruments has become alarmingly widespread, while the understanding of how well they work and how accurate and reliable they are has dwindled significantly. This trend was first pointed out with respect to the use of computer mice by Beringer (1992), more recently described by Plant et al. (2002, 2003), then by Plant et al. (2004), and then again in 2009 (Plant and Turner), the latter after the use of mobile “smart” devices had begun its exponential rise. In their 2009 paper, Plant and Turner updated their earlier findings and observed that the trend had not improved. They noted that millisecond *precision* is a very different thing from millisecond *accuracy*. Even with newer human interface technologies, timing accuracy has not enjoyed the same priority and improvements over time as cost reduction. They conclude that, “It is important to note that the fact that hardware and software produce answers that “look accurate” does not mean that those answers are valid.”

In 2016 Plant (Plant, 2016) again emphasizes that millisecond timing accuracy errors are prevalent throughout the psychology literature and that this may contribute to what is now recognized as the “replication crisis.” The replication crisis appears to span most of biomedical research, even in cancer and drug development research (Prinz et al., 2011; Begley and Ellis, 2012) in which careful replication exercises demonstrate that as much as 75 to 89% of academic research, published by the best laboratories in top journals, may simply not be reproducible. Many factors contribute to the replication crisis across medical research. Plant suggests that within the field of psychology, this is likely due in part to hardware and software problems that contribute to timing errors and reproducibility problems between different laboratories. Plant further points out that faster hardware has not improved timing accuracy, rather over the years it has apparently gotten worse, and that most researchers simply do not know what their timing accuracy actually is. Further complicating the issue is the fact that web-based studies have become increasingly common and have introduced several new sources of inaccuracy, including server load and caching of scripts. Plant states, “In sum, accuracy has continued to decrease but our confidence in the equipment and the perception of accuracy has risen as computers have become faster and ubiquitous.” The significance of this for concussion research is that the inaccuracies introduced to reaction time measures could make it difficult to replicate studies. A study conducted on one computer system will introduce both latency and variability errors—some of which will be unique to the computer system that the online testing is being run on. Even if the computer systems are replicated from one study site to another, the variability of the system will contribute to inaccuracies in the study and make it difficult to replicate. Additionally, these same errors make the reaction time measure untrustworthy as a clinical outcome measure (which could also be significantly different for an individual testing at multiple locations), and it makes reaction time variability not viable as an individual outcome measure. Although group averages will continue to show general trends (i.e., concussed individuals having reaction times slower than non-concussed individuals) with sufficiently high numbers of subjects that yield statistical significance, an individual measure of reaction time will simply not have the necessary reliability to be counted on as a clinical metric. If the goal of clinical research is to develop and/or improve outcome measures to monitor an individual's status, then inaccurate measures that are not replicable are a disservice to all. From a broader perspective, systematic instrumentation errors that result from the misuse and misunderstanding of basic research test equipment leads directly to one and/or two outcomes: (1) data with significant absolute errors, which may or may not be internally replicable enough to allow the use of data variability as a useful metric, and subsequently adds more erroneous data to the body of peer-reviewed literature, and (2) lack of awareness on the part of authors of such systematic errors, which leads to erroneous or under-reporting of details of the experimental apparatus used to inaccurately collect the data. Thus, by adding inaccurate data and inadequate methodological detail, the replicability crisis is exacerbated, since the data could not be reproduced independently. Systematic errors of this type lead both to the publication of bad data, and to the wider problem of scientific reproducibility.

For a scientific measure to be valid, it must be both accurate and precise. The problem with commodity-grade computer interfaces is that they may be precise while not being accurate, or they may lack both precision and accuracy because they typically introduce constant or variable non-zero timing offset biases that cannot simply be overcome by “taking more samples” and relying upon the central limit theorem as suggested by Ulrich and Giray (Ulrich and Giray, 1989). Using this approach with any form of systematic bias, additional samples will only render a result that is more precisely inaccurate. The paradox faced by researchers is that while modern commodity-grade human computer interface devices and networking increasingly gain the veneer of smooth glitch-less operation, precise timing tasks in the background are compromised in increasingly subtle ways that are more difficult to detect, quantify, predict, and eliminate.

Could improvements to the accuracy and precision of reaction time testing increase the reliability of computerized assessments? In the authors' opinion, this is a resounding yes. To address this question, consider the example of mild traumatic brain injury (mTBI), which is just one of many neurological disorders that demonstrate an altered reaction time. Multiple reports have noted the importance of reaction time assessment in monitoring mTBI and concussion (Ruesch, 1944; van Zomeren and Deelman, 1976; MacFlynn et al., 1984; Stuss et al., 1989; Ponsford and Kinsella, 1992; Collins and Long, 1996; Zahn and Mirsky, 1999; Warden et al., 2001; Collins et al., 2003; Sarno et al., 2003; Willison and Tombaugh, 2006; Niogi et al., 2008; Gould et al., 2013; Eckner et al., 2015). More recently, investigators have recognized that reaction time variability is a better indicator for cognitive function than reaction time alone, suggesting that it is much more sensitive to neurological disorders such as concussion (Cole et al., 2018).

The evaluation of individuals who have sustained mild traumatic brain injury has been growing in prominence in the public forum, with much of this debate arising from the widespread inadequacy of the methods commonly used to assess cognitive function and the neurological insults that are caused by mTBI. One of the measures that is commonly obtained by most online cognitive assessment tools is simple reaction time, yet very few of these online assessment tools have the capacity to evaluate the metric accurately, much less the capacity to evaluate reaction time variability. Reaction time variability has a normative range of 10–20 msec, which simply cannot be measured by systems that have variable latency ranges of 84 msec. Normative reaction time is in the 200–220 msec range (Zhang et al., 2011; Favorov et al., 2019; Pearce et al., 2019), so introduction of this amount of error is also significant. Additionally, many of these assessments are performed on multiple computers and operating systems for the same subject, which can lead to errors caused by inconsistencies between different systems. As mentioned above, reaction time variability appears to be a more important measure for mTBI assessment than reaction time, and given the extreme sensitivity of this measure to the timing errors introduced by commodity grade computing, the need for improved accuracy is significant.

### How Good an Outcome Measure Is Reaction Time Testing for Concussion?

**Figure 7:**
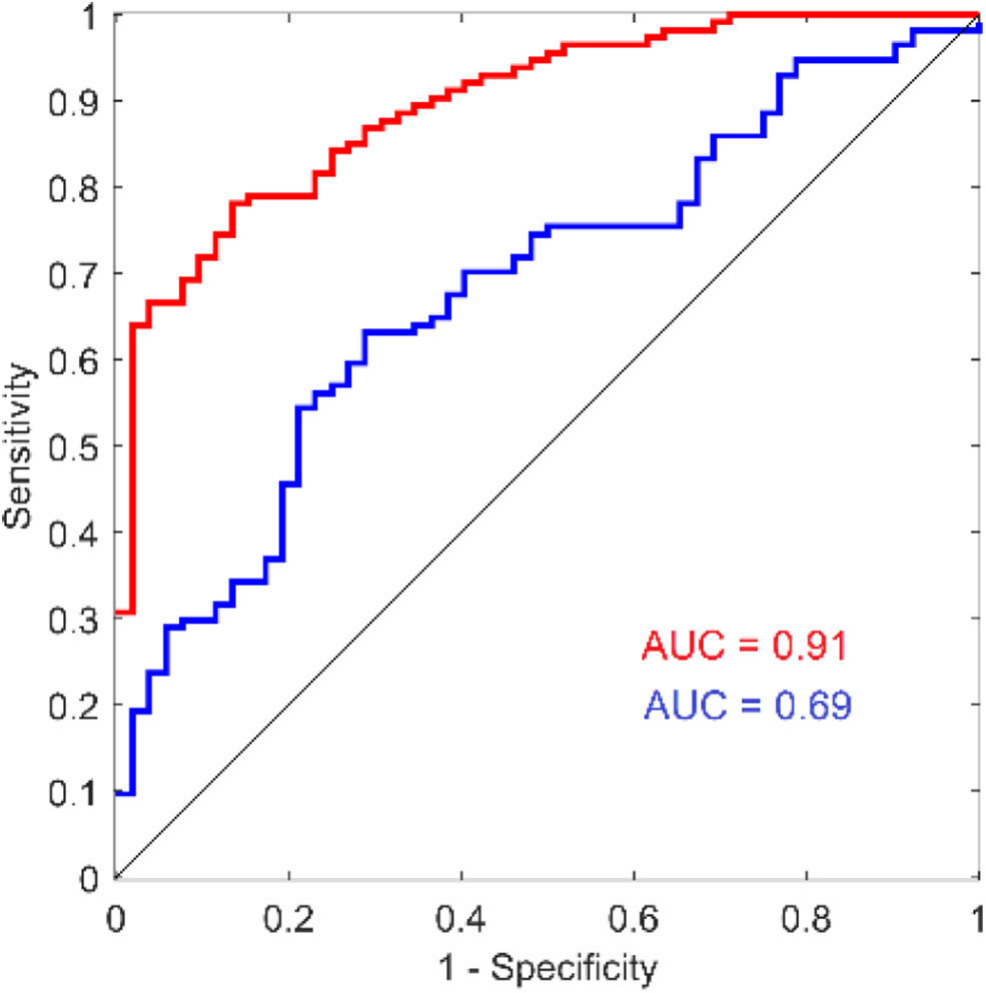
ROC curves for reaction time and reaction time variability from a recent study comparing concussed to non-concussed individuals. Reproduced with modification and with permission from (Favorov et al., 2019).

Although there have been numerous reports that used reaction time as an outcome measure for concussion studies, the results & conclusions from those findings vary widely. Some reports described little or no difference between the reaction time of concussed and non-concussed individuals (Iverson et al., 2004; Straume-Naesheim et al., 2005), and some have even reported that concussed individuals have faster reaction times than non-concussed individuals (Lynall et al., 2018; Iverson et al., 2019; Norman et al., 2019). In one study, comparison of reaction time measures showed no correlation between the metric obtained with several different computerized methods (Schatz and Putz, 2006) even though the methods were all targeting simple reaction time from the same cohort. The authors' interpretation of these findings is that they reflect what could happen with poorly administered and inaccurate testing methods. On the other hand, a number of reports have demonstrated utility for reaction time as a concussion assessment (Warden et al., 2001; Willison and Tombaugh, 2006; Cole et al., 2018; Danna-Dos-Santos et al., 2018; Lange et al., 2018). Norris et al. (2013) described the reaction time metric as having prognostic utility and others have proposed that reaction time could be used in the absence of baseline measures by just comparing to normative values (Schmidt et al., 2012), and the authors share that opinion based on our recent work. For example, note the ROC curve in Figure 7 (reproduced from Favorov et al., 2019) that shows the area under the receiver operating characteristic (ROC) curve to be 0.69 for predicting concussed status based on tactile reaction time with the same configuration described in the methods section in this report. While this is not a perfect outcome measure by itself, it indicates good sensitivity and specificity and makes a good argument to use the reaction time metric as an aid in the assessment of concussed individuals. More significantly, the reaction time variability metric (also referenced as intraindividual variability in some reports and an obvious by-product of collecting reaction time), appears to be a very good predictor of concussion in the cohort studied (note AUC of 0.91). Other investigators have also pointed out that reaction time variability could be an extremely useful tool in assessments of neurological disorders (Collins et al., 1999; MacDonald et al., 2003, 2006) including concussion (Cole et al., 2018). Interestingly, one report stated that although this variability had been demonstrated in a number of reports to be a good indicator of neurological dysfunction, it stated that concussion was not in the same category as other brain insults because reaction time variability was not altered in the concussed individuals that they studied (Sosnoff et al., 2007). The methods of that report stated that an online cognitive test was administered on a computer—no details as to resolution were given—and given the results of this report, the authors suspect that the resolution of that experiment was inadequate to obtain results accurate enough to evaluate reaction time variability.

There has been a growing interest in the long-term impact of concussions and the symptoms that often linger post-concussion. Research into persistent post-concussion symptoms (PPCS) has mostly investigated the ongoing effects on cognitive functioning, such as working memory, attention and concentration, and executive functioning. However, interest in the sensory-motor system in quantifying ongoing concussion symptoms has emerged in the last decade (De Beaumont et al., 2007, 2011; Johansson et al., 2009; Pearce et al., 2014, 2015), and accurate measures of reaction time and reaction time variability make it possible to detect an altered state in this population. Recently we demonstrated in people with PPCS that those with ongoing self-reported symptoms had significantly slower reaction times and had significantly higher reaction time variabilities compared to both those who had fully recovered from a concussion and age-matched healthy controls (Pearce et al., 2019). Additionally, the study reported that reaction time variability co-varied with fatigue of the individuals with PPCS and thus highlights the impact that a sensitive measure could have in post-concussion assessments.

Although this report focuses on the technological differences in commercially available tactile vs. visual methodology in reaction time testing, it does prompt speculation on the differences that *would be based* on biological differences. In this paper, we did not use any data from human subjects, so that those differences would not have impact on the results. However, there have been multiple publications that examined the results from reaction time testing with the tactile based approach described here, and these results demonstrated that reaction time and reaction time variability can be very useful for testing concussed individuals. Favorov et al. (2019) demonstrated that reaction time testing with the Brain Gauge can differentiate concussed from non-concussed individuals: using the ROC (Receiver Operating Characteristic) curve analysis, they found the area under the curve AUC = 91% for reaction time variability and AUC = 69% for reaction time, which suggests that reaction time variability is much more sensitive to that condition. Similarly, another study showed reaction time and reaction time variability (with the Brain Gauge) to differentiate 3 groups of individuals—healthy controls, individuals that had recovered from concussion and individuals with chronic symptoms of concussion (Pearce et al., 2019). Data from a large cohort of individuals with sports concussion were examined and also demonstrated that differences in mean values of reaction time and reaction time variability (with the Brain Gauge) are statistically highly significant between healthy control and post-concussion populations (*p* < 10^−15^; Tommerdahl et al., 2020). The difference in reaction times of the tested groups was ~30% while the difference in reaction time variability was over 80%, again strongly suggesting that reaction time variability is a much more sensitive measure for detecting alterations of information processing speed due to concussion than reaction time. Reports of visual reaction time testing have simply not demonstrated such levels of discriminability, which leads to the question: how do tactile based results with the Brain Gauge directly compare with those obtained from visual reaction time testing? Using the same methods described in this report (that were done robotically), comparison of tactile reaction time with the Brain Gauge was compared with visual reaction time with healthy controls. From the data presented in this paper, the expected difference in the tactile vs. visual reaction times should be ~80 msec, and the results in the study with healthy controls was slightly over that value (~85 msec; Kim et al., 2020), and the measured reaction time variability for that cohort was 16 msec vs. 81 msec (tactile vs. visual). This finding leads to the next question: is that difference in visual vs. tactile reaction time predictive of findings in other studies? In a separate study that compared observations from individuals with persistent post-concussion symptoms (PPCS) and healthy controls with both tactile reaction time (collected with the Brain Gauge) and visual reaction time (collected with CogState, a commercial program), there was a difference in the mean visual reaction time and the mean tactile reaction time of the healthy controls of slightly over 80 msec (Pearce et al., 2020). Does this mean that subtracting the delay introduced with the visual reaction time task will then provide an accurate measure? The answer is a resounding no. The increased variability of the visual reaction time measure—which is effectively random—leads to inaccuracies that make it ineffective. In that same study, there was a statistically significant difference of the tactile reaction time collected from healthy controls and individuals with PPCS, but there was no difference detected with the visual reaction time. There was also a significant difference with tactile reaction time variability, but the visual reaction time variability could not be evaluated.

It is possible that the differences in tactile vs. visual reaction times could be partially accounted for by biological differences, but it appears to be, from the authors point of view, primarily technology based. Two of the authors (Oleg Favorov and Mark Tommerdahl) had several discussions with the late Steve Hsiao (Johns Hopkins University) in the 1990s about differences between the somatosensory and visual systems. Steve was of the opinion that object recognition takes place in different sensory modalities (such as touch and vision), but projects to the same decision center (Hsiao, 1998), and any biological differences between visual and tactile reaction time should be limited. This may seem surprising to many, simply because most reported visual reaction times are slower than reported tactile reaction times (e.g., the afore mentioned Pearce et al., 2020 study reported that visually based reaction times were 95 msec slower than tactile times for healthy controls), and based on the data presented in this report, the differences in reaction time that are often reported between visual and tactile are most likely methodological. Technological differences aside, the tactile component of the somatosensory system may also be a better vehicle for delivering reaction time testing and probably affords a vehicle for delivering better assessments and research results with lower inter-subject variability. From an engineering perspective, there is a much better signal-to-noise ratio for inputs through the somatosensory system to be much higher fidelity simply because of the lack of environmental noise. Inputs via somatosensation do not have to compete with other inputs, which is in sharp contrast to the testing environment that most visual tests contend with. As pointed out by Leonard (1959), “.stimulation of the fingers seems to be the answer” to obtaining an accurate measure of pure reaction time with minimal distractions. Although Leonard was addressing a strategy aside from the methodological problems that were not introduced until decades after his experiments when consumer grade technology became prevalent in research, his conclusions were quite prescient, and future studies will expand on this concept.

## Conclusions

The results suggest that some of the commonly used visual tasks on consumer grade computers could be (and have been) introducing significant errors for reaction time testing and that dedicated hardware designed for the reaction time task is needed to minimize testing errors.

## Data Availability Statement

The datasets generated for this study are available on request to the corresponding author.

## Author Contributions

MT and JH conceived the idea behind manuscript. LZ provided overall guidance of the design of the manuscript. EF and RL analyzed the data and generated the figures. BK built supporting hardware for the project. JH wrote the software. BK and JH wrote the methods and results section. BK, EF, and JH conducted the experiments. AT provided background research and provided significant input to the introduction and discussion. RGD and MT wrote the introduction and discussion. AJP and OVF provided important contributions to the discussion.

## Conflict of Interest

JH, EF, AT, RL, BK, RD, and MT were employed by company Cortical Metrics LLC. LZ was employed by company Lucent Research. The remaining authors declare that the research was conducted in the absence of any commercial or financial relationships that could be construed as a potential conflict of interest.

## Abbreviations

CPU: Central Processing Unit
VCA: Voice Coil Actuator
LCD: Liquid Crystal Display
IQ: Intelligence Quotient
FET: Field Effect Transistor
msec: milliseconds
mTBI: mild traumatic brain injury
USB: Universal serial bus.
