# Supplementary material for: Methodological Problems With Online Concussion Testing
Source: Front Hum Neurosci. 2020 Oct 1;14:509091. doi: 10.3389/fnhum.2020.509091 (PMC7559397; doi:10.3389/fnhum.2020.509091)
Supplement: Supplementary file 1 [file Table_1.pdf]

| Ref | Author | Year | Country | Age Range | Visual RT (ms) | Auditory RT (ms) | Tactile RT (ms) |
|---|---|---|---|---|---|---|---|
| 2 | Anger et al. | 1993 | USA | 16-25 | 275.9 | | |
| 2 | Anger et al. | 1993 | USA | 26-35 | 270.7 | | |
| 3 | Arab & Ang | 2015 | USA | 20-59 | 242 | | |
| 5 | Benton | 1977 | USA | 16-44 | 220 | 190 | |
| 5 | Benton | 1977 | USA | 46-63 | 240 | 215 | |
| 7 | Blackburn & Benton | 1955 | USA | mean=38.5 | 208 | | |
| 8 | Brice & Smith | 2002 | UK | 19-23 | 324 | | |
| 9 | Burpee & Stroll | 1936 | USA | [not given] | 154 | | |
| 10 | Cattell | 1886 | Germany | [not given] | 180 | | 155 |
| 13 | Collingnon & De Volder | 2009 | Canada | mean: 30±10 | | 380 | 350 |
| 14 | Collins & Long | 1996 | USA | 18-46 | 310 | | |
| 16 | Davidson et al. | 1990 | USA | mean 11.18±1.11 | 320 | | 345 |
| 17 | Deary and Der | 2005 | UK | [not given] | 300.3 | | |
| 18 | Der & Deary | 2006 | UK | 18-42 | 309.6 | | |
| 20 | Donders | 1868 | Germany | "generally young" | | 201 | |
| 22 | Edwards & Cohen | 1961 | USA | 24-48 | 234.47 | | |
| 23 | Edwards et al. | 2007 | UK | 35±1.3 | 276 | 221 | 296 |
| 24 | Evarts et al. | 1981 | USA | 20-45 | 254 | | |
| 24 | Evarts et al. | 1981 | USA | 52-80 | 308 | | |
| 26 | Forbes | 1945 | UK | 17-53 | 286 | | |
| 27 | Fozard et al. | 1994 | USA | 16-24 | | 225 | |
| 27 | Fozard et al. | 1994 | USA | 25-34 | | 250 | |
| 27 | Fozard et al. | 1994 | USA | 35-44 | | 250 | |
| 27 | Fozard et al. | 1994 | USA | 45-54 | | 260 | |
| 27 | Fozard et al. | 1994 | USA | 55-64 | | 260 | |
| 27 | Fozard et al. | 1994 | USA | 65-74 | | 260 | |
| 27 | Fozard et al. | 1994 | USA | 75-84 | | 275 | |
| 27 | Fozard et al. | 1994 | USA | 85+ | | 265 | |
| 28 | Galton | 1880 | UK | [not given] | 185 | | |
| 29 | Goodenough | 1935 | USA | "college aged" | | 172 | |
| 30 | Goodrich et al. | 1989 | UK | 57-79 | | | 215 |
| 31 | Gould et al. | 2013 | USA | 22-27 | 279 | | |
| 32 | Hashemi | 2015 | Iran | 25+ | 371.2 | | |
| 36 | Hyman | 1953 | USA | 18-22 | 200 | | |
| 34 | Hetherington, Stuss, & Finlayson | 1996 | Canada | 15-60 | 250 | | |
| 35 | Hick | 1952 | UK | experimenter + research worker | 170 | | |
| 37 | Johanson | 1922 | USA | [not given] | | 143.9 | |
| 38 | Jones | 1937 | USA | 9th grade | 151.4 | | |
| 39 | Jorm et al. 2004 | 1999 | Australia | 20-24 | 219.5 | | |
| 39 | Jorm et al. 2004 | 1999 | Australia | 40-44 | 232.8 | | |
| 39 | Jorm et al. 2004 | 1999 | Australia | 60-64 | 257.5 | | |
| 40 | Kleitman et al. | 1937 | USA | graduate students + instructor | 163 | 153 | |
| 41 | Klemmer | 1957 | USA | 2 college students + 3 lab workers | 170 | | |
| 42 | Kumar et al. | 2010 | India | 7-May | 260 | 256 | 270 |
| 42 | Kumar et al. | 2010 | India | 10-Aug | 253 | 245 | 267 |
| 42 | Kumar et al. | 2010 | India | 12-Nov | 248 | 233 | 252 |
| 42 | Kumar et al. | 2010 | India | 13-15 | 236 | 225 | 237 |
| 42 | Kumar et al. | 2010 | India | 18-20 | 227 | 204 | 221 |
| 42 | Kumar et al. | 2010 | India | 21-25 | 207 | 181 | 213 |
| 42 | Kumar et al. | 2010 | India | 26-30 | 213 | 195 | 214 |
| 42 | Kumar et al. | 2010 | India | 31-35 | 234 | 225 | 238 |
| 42 | Kumar et al. | 2010 | India | 36-40 | 260 | 240 | 264 |
| 42 | Kumar et al. | 2010 | India | 41-45 | 263 | 265 | 277 |
| 42 | Kumar et al. | 2010 | India | 46-50 | 285 | 276 | 289 |
| 42 | Kumar et al. | 2010 | India | 51-55 | 300 | 283 | 307 |
| 42 | Kumar et al. | 2010 | India | 56-60 | 305 | 290 | 313 |
| 42 | Kumar et al. | 2010 | India | 61-65 | 309 | 297 | 320 |
| 42 | Kumar et al. | 2010 | India | 66-70 | 328 | 320 | 336 |
| 44 | Lefcourt & Siegel | 1970 | Canada | "college aged" | 249.5 | | |
| 45 | Leonard | 1959 | UK | [not given] | | | 182 |
| 46 | Lorenzo et al. | 1995 | Mexico | 22-30 | 160 | | |
| 49 | Merkel | 1885 | Germany | [not given] | 190 | | |
| 53 | Obersteiner | 1874 | Germany | [not given] | 140 | | |
| 54 | Patil & Phatale | 2015 | India | 40-50 | 220 | 176 | |
| 60 | Ponsford & Kinsella | 1992 | Australia | 16-43 | 410 | | |
| 62 | Puts et al. | 2014 | USA | 12-Aug | | | 310.79 |
| 63 | Reed, Vernon, & Johnson | 2004 | Canada | 18-25 | 261 | | |
| 64 | Richerson, Robinson & Shum | 2005 | USA | mean: 22.7±1.2 | | 218.6 | 216 |
| 64 | Richerson, Robinson & Shum | 2005 | USA | mean: 59.4±8 | | 276.9 | 331.5 |
| 65 | Ruesch | 1944 | USA | mean: 28.6±8.4 | 300 | | |
| 66 | Sarno et al. | 2003 | Germany | 17-66 | 242 | 200 | 291 |
| 67 | Schilling | 1920 | USA | [not given] | | 166 | |
| 69 | Seashore et al. | 1941 | USA | "college aged" | 203 | | |
| 69 | Seashore et al. | 1941 | USA | "college aged" | 197 | | |
| 70 | Sherwood & Selder | 1979 | USA | 20-29 | 185 | | |
| 70 | Sherwood & Selder | 1979 | USA | 30-39 | 195 | | |
| 70 | Sherwood & Selder | 1979 | USA | 30-39 | 205 | | |
| 70 | Sherwood & Selder | 1979 | USA | 40-49 | 195 | | |
| 70 | Sherwood & Selder | 1979 | USA | 40-49 | 207 | | |
| 70 | Sherwood & Selder | 1979 | USA | 50-59 | 190 | | |
| 70 | Sherwood & Selder | 1979 | USA | 50-59 | 227 | | |
| 71 | Smith et al. | 1999 | UK | "college aged" | 306 | | |
| 72 | Spence et al. | 2000 | Australia | 17-49 | 300 | 270 | 260 |
| 73 | Stuss et al. | 1989 | Canada | 16-20 | 243.1 | | |
| 73 | Stuss et al. | 1989 | Canada | 16-54 | 237.4 | | |
| 73 | Stuss et al. | 1989 | Canada | 16-63 | 228.1 | | |
| 74 | Taimela | 1991 | Finland | 17-29 | 199 | | |
| 74 | Taimela, Kujala, & Osterman | 1991 | Finland | 18-28 | 183 | | |
| 76 | Thompson | 1885 | USA | [not given] | 208 | | |
| 77 | Tuttle & Brown | 1926 | USA | [not given] | | 164.3 | |
| 79 | Van Zomeren & Deelman | 1976 | Netherlands | 16-39 | 259 | | |
| 80 | Warden et al. | 2001 | USA | "youngish" | 254 | | |
| 81 | Wells | 1913 | USA | [not given] | 180 | | |
| 82 | Westerlund & Tuttle | 1931 | USA | "college aged" | 152 | | |
| 83 | Willison & Tombaugh | 2006 | Canada | mean: 19.5±1.22 | 284.61 | | |
| 85 | Yazdi et al. | 2011 | Iran | mean: 37.71±5.22 | 270.89 | | |
| 86 | Zahn & Mirsky | 1999 | USA | 18-56 | | 230 | |

**Table 2.**

| Modality | Average Latency | Range of Latencies |
|---|---|---|
| Visual RT + Touchscreen | 399 ± 16.3 | 348 - 432 |
| Visual RT + Mouse | 80.1 ± 8.0 | 59 - 104 |
| Tactile RT + Mouse | 31.7 ± 2.6 | 27 - 37 |
| Tactile RT + BG | 5.6 ± 0.25 | 4.8 - 6.1 |

**References**


Anger WK, Cassitto MG, Liang YX, Amador RA, Hooisma JA, Chrislip DW, et al. (1993). Comparison of performance from three continents on the WHO-recommended neurobehavioral core test battery. Environmental Research. 62, 125-147.

Arab L, Ang A. (2015). A cross sectional study of the association between walnut consumption and cognitive function among adult us populations represented in NHANES. The journal of nutrition, health & aging. 19, 284-290.

Benton AL. (1977). Interactive effects of age and brain disease on reaction time. Archives of Neurology. 34, 369-370.

Blackburn HL, Benton AL. (1955). Simple and choice reaction time in cerebral disease. Stereotactic and Functional Neurosurgery.15, 327-338.

Brice CF, Smith AP. (2002). Effects of caffeine on mood and performance: a study of realistic consumption. Psychopharmacology. 164, 188-192.

Burpee RH, Stroll W. (1936). Measuring reaction time of athletes. Research Quarterly. American Physical Education Association. 7, 110-118.

Cattell JM. (1886). The influence of the intensity of the stimulus on the length of the reaction time. Brain. 8, 512-515.

Collignon O, De Volder AG. (2009). Further evidence that congenitally blind participants react faster to auditory and tactile spatial targets. Canadian Journal of Experimental Psychology/Revue canadienne de psychologie expérimentale. 63, 287.

Collins LF, Long CJ. (1996). Visual reaction time and its relationship to neuropsychological test performance. Archives of Clinical Neuropsychology. 11, 613-623.

Davidson RJ, Leslie SC, Saron C. (1990). Reaction time measures of interhemispheric transfer time in reading disabled and normal children. Neuropsychologia. 28, 471-485.

Deary IJ, Der G. (2005). Reaction time, age, and cognitive ability: Longitudinal findings from age 16 to 63 years in representative population samples. Aging, Neuropsychology, and cognition. 12, 187-215.

Der G, Deary IJ. (2006). Age and sex differences in reaction time in adulthood: results from the United Kingdom Health and Lifestyle Survey. Psychology and Aging. 21, 62.

Donders FC. (1969). On the speed of mental processes. Acta psychologica. 30, 412-431.

Donders FC. (1868). Over de snelheid van psychische processen. Onderzoekingen gedaan in het Physiologisch Laboratorium der Utrechtsche Hoogeschool. 2, 92-120.

Edwards AE, Cohen S. (1961). Visual illusion, tactile sensibility and reaction time under LSD-25. Psychopharmacologia. 2, 297-303.

Edwards L, Ring C, McIntyre D, Carroll D, Martin U. (2007). Psychomotor speed in hypertension: Effects of reaction time components, stimulus modality, and phase of the cardiac cycle. Psychophysiology. 44, 459-68.

Evarts EV, Teräväinen H, Calne DB. (1981). Reaction time in Parkinson's disease. Brain: a journal of neurology. 104, 167-186.

Forbes G. (1945). The effect of certain variables on visual and auditory reaction times. Journal of Experimental Psychology. 35, 153.

Fozard JL, Vercruyssen M, Reynolds SL, Hancock PA, Quilter RE. (1994). Age differences and changes in reaction time: the Baltimore Longitudinal Study of Aging. Journal of gerontology. 49, 179-189.

Galton F. (1890). Mental tests and measurements. Mind. 15, 373-381.

Goodenough FL. (1935). The development of the reactive process from early childhood to maturity. Journal of Experimental Psychology. 18, 431.

Goodrich S, Henderson L, Kennard C. (1989). On the existence of an attention-demanding process peculiar to simple reaction time: converging evidence from Parkinson's disease. Cognitive Neuropsychology. 6, 309-331

Gould JA, Ciuffreda KJ, Yadav NK, Thiagarajan P, Arthur B. (2013). The effect of retinal defocus on simple eye-hand and eye-foot reaction time in traumatic brain injury (TBI). Brain injury. 27, 1643-1648.

Hashemi F, Ashtiani AF, Mirminachi B, Sharafkhah M, Ekhlasi G, Jafari E, et al. (2015). Impact of hepatitis C virus infection on cognitive function in patients with covert hepatic encephalopathy. Hepatitis monthly. 15, 7.

Hetherington CR, Stuss DT, Finlayson MA. (1996). Reaction time and variability 5 and 10 years after traumatic brain injury. Brain injury. 10, 473-486.

Hick WE. (1952). On the rate of gain of information. Quarterly Journal of Experimental Psychology. 4, 11-26.

Hyman R. (1953). Stimulus information as a determinant of reaction time. Journal of experimental psychology. 45, 188.

Johanson AM. (1922). The influence of incentive and punishment upon reaction-time. Archives of psychology. 8, 53.

Jones HE. (1937). Reaction-time and motor development. The American Journal of Psychology. 50, 181-94.

Jorm AF, Anstey KJ, Christensen H, Rodgers B. (2004). Gender differences in cognitive abilities: The mediating role of health state and health habits. Intelligence. 32, 7-23.

Kleitman N, Titelbaum S, Feiveson P. (1938). The effect of body temperature on reaction time. American Journal of Physiology-Legacy Content. 121, 495-501.

Klemmer ET. (1957). Simple reaction time as a function of time uncertainty. Journal of experimental psychology. 54, 195.

Kumar BA, Shikha D, Sudarsan B. (2010). Reaction time with respect to the nature of stimulus and age of male subjects. Journal of sport and health research. 2, 35-40.

Lefcourt HM, Siegel JM. (1970). Reaction-time behaviour as a function of internal-external control of reinforcement and control of test administration. Canadian Journal of Behavioural Science/Revue canadienne des sciences du comportement. 2, 253.

Leonard JA. (1959). Tactual choice reactions: I. Quarterly Journal of Experimental Psychology. 11, 76-83.

Lorenzo I, Ramos J, Arce C, Guevara MA, Corsi-Cabrera M. (1995). Effect of total sleep deprivation on reaction time and waking EEG activity in man. Sleep. 18, 346-354.

Merkel J. (1885). Die zeitlichen  verhaltnisse der willensthatigkeit. Philos Stud. 2, 73-127.

Obersteiner H. (1874). Über eine neue einfache Methode zur Bestimmung der psychischen Leistungsfähigkeit des Gehirnes Geisteskranker. Virchows Archiv. 59, 427-458.

Patil S, Phatale S. (2015). Auditory and Visual Reaction Time-A Tool for Early Detection of Neuropathy in Diabetics. International Journal of Health Sciences and Research. 5, 141-146.

Ponsford J, Kinsella G. (1992). Attentional deficits following closed-head injury. Journal of Clinical and Experimental Neuropsychology. 14, 822-838.

Puts NA, Wodka EL, Tommerdahl M, Mostofsky SH, Edden RA. (2014). Impaired tactile processing in children with autism spectrum disorder. Journal of Neurophysiology. 111, 1803-1811.

Reed TE, Vernon PA, Johnson AM. (2004). Sex difference in brain nerve conduction velocity in normal humans. Neuropsychologia. 42, 1709-1714.

Richerson SJ, Robinson CJ, Shum J. (2005). A comparative study of reaction times between type II diabetics and non-diabetics. Biomedical engineering online. 4, 12.

Ruesch J. (1944). Dark adaptation, negative after images, tachistoscopic examinations and reaction time in head injuries. Journal of Neurosurgery. 1, 243-251.

Sarno S, Erasmus LP, Lipp B, Schlaegel W. (2003). Multisensory integration after traumatic brain injury: a reaction time study between pairings of vision, touch and audition. Brain Injury. 17, 413-426.

Schilling W. (1921). The effect of caffeine and acetanilide on simple reaction time. Psychological Review. 28, 72.

Seashore RH, Starmann RA, Kendall WE, Helmick JS. (1941). Group factors in simple and discriminative reaction times. Journal of Experimental Psychology. 29, 346.

Sherwood DE, Selder DJ. (1979). Cardiorespiratory health, reaction time and aging. Medicine and science in sports. 11, 186-189.

Smith A, Sturgess W, Richi N, Brice C, Collisonl C, Bailey J, et al. (1999). The effects of idazoxan on reaction times, eye movements and the mood of healthy volunteers and patients with upper respiratory tract illnesses. Journal of psychopharmacology. 13, 148-151.

Spence C, Lloyd D, McGlone F, Nicholls ME, Driver J. (2000). Inhibition of return is supramodal: a demonstration between all possible pairings of vision, touch, and audition. Experimental Brain Research. 134, 42-48.

Stuss DT, Stethem LL, Hugenholtz H, Picton T, Pivik J, Richard MT. (1989). Reaction time after head injury: fatigue, divided and focused attention, and consistency of performance. Journal of Neurology, Neurosurgery & Psychiatry. 52, 742-748.

Taimela S, Kujala UM, Osterman K. (1991). The relation of low grade mental ability to fractures in young men. International orthopaedics. 15, 75-77.

Tamm L, Narad ME, Antonini TN, O'Brien KM, Hawk LW, Epstein JN. (2012). Reaction time variability in ADHD: a review. Neurotherapeutics. 9, 500-508.

Thompson HB. (1905). The mental traits of sex: An experimental investigation of the normal mind in men and women. Chicago: The University of Chicago Press.

Tuttle WW, Brown, TT. (1926). Studies in reaction time, American Journal of Physiology. 78, 150-157.

Van Zomeren AH, Deelman BG. (1976). Differential effects of simple and choice reaction after closed head injury. Clinical Neurology and Neurosurgery. 79, 81-90.

Warden DL, Bleiberg J, Cameron KL, Ecklund J, Walter J, et al. (2001). Persistent prolongation of simple reaction time in sports concussion. Neurology. 57, 524-6.

Wells GR. (1913). The influence of stimulus duration on reaction time. The Psychological Monographs. 5, i.

Westerlund JH, Tuttle WW. (1931). Relationship between running events in track and reaction time. Research Quarterly. American Physical Education Association. 2, 95-100.

Willison J, Tombaugh TN. (2006). Detecting simulation of attention deficits using reaction time tests. Archives of Clinical Neuropsychology. 21, 41-52.

Yazdi SM, Sharifian A, Dehghani-Beshne M, Momeni VR, Aminian O. (2011). Effects of fluoride on psychomotor performance and memory of aluminum potroom workers. Fluoride. 44, 158-62.

Zahn TP, Mirsky AF. (1999). Reaction time indicators of attention deficits in closed head injury. Journal of Clinical and Experimental Neuropsychology. 21, 352-67.
